# Medical News and Miscellany

**Published:** 1894-11

**Authors:** 


### Medical News and Miscellany.

---

**Dr. T. N. Skeen** has removed from Wichita Falls to Winsboro, Texas.

---

**Dr. Rudolph Matas** has been appointed Professor of Surgery of Tulane University Medical College vice the lamented Miles.

---

**Wanted.**—A copartnership with a physician doing a paying and growing practice in a growing town. Address Dr. W., care TEXAS MEDICAL JOURNAL, Austin, Texas.

---

**For Sale.**—Practice of $2,000, on railroad. Small village, good churches and schools. Will sell residence and give practice and good will. Address R. H., care TEXAS MEDICAL JOURNAL, Austin, Texas.

---

**One Hundred and Seventy-eight!**—That is the figure reached on the matriculation list of the Medical Department, Texas University, and this its third year. We *told* you so. Excelsior is the word, and the top is the goal! No college will turn out better prepared doctors than the Texas Medical College.

---

**Prof. J. S. Cain, M. D.,** whose resignation from the Nashville Medical College Faculty, in which he had for sixteen years held the Chair of Practice, we noted last month, we are pleased to see has returned to the old college and taken the Chair of Principles of Surgery, made vacant by the resignation of Dr. Duncan Eve. The Sewanee Medical College (Summer) is not affected by this change.

---

**Official Organ.**—The Houston District Medical Association, by resolution adopted October 22d ult., declared the TEXAS MEDICAL JOURNAL the official organ of that Society, and notice to that effect was furnished the JOURNAL by its courteous secretary, Dr. J. B. Massie. We will have many good papers from them for the benefit of the readers of the "Red Back," and to begin with, we present one this month from the President, Dr. Cook.

---

**For Sale.**—Residence and practice in a flourishing small town in the German and Bohemian settlement, where practice is mostly cash. Business about $2000 a year. House of 5 rooms, situated on a block of ground, good out houses. splendid barn and good fruit orchard. Price, $1000, half cash. The money can be made out of the practice the first year, by a good doctor. Reason for selling, to remove to a city. For particulars address E. R. W., care TEXAS MEDICAL JOURNAL, Austin, Texas.

**Kind Words.**—The TEXAS MEDICAL JOURNAL, published at Austin, with its attractive red dress, chapters of indispensable medical knowledge and its enviable array of patrons, comes to our table a most welcome visitor. It is a fitting example to the profession of what can be accomplished by masterly management and good endeavor. The JOURNAL has few peers, East or West.—*From N. A. Medical Review.*

---

**Mississippi Valley Medical Association.**—For the Mississippi Valley Medical Association meeting at Hot Springs, Ark., November 20th to 23d, the International Route, I. & G. N. R. R., will make rate of one fare for round trip from all points on its line to Hot Springs and return. Tickets on sale November 17th to 20th inclusive, and limited to 20 days for return.

Call on nearest ticket agent for full information, or address

D. J. PRICE, A. G. P. A.,
Palestine, Texas.

---

**Hymeneal.**—Two doctors with but a single thought, two practices that count as one. Our esteemed fellow citizen and long time practitioner Dr. W. J. Mathews will on the 15th inst., God willing, be united in marriage to Dr. Florence E. Collins, late of Austin, and formerly of Wisconsin. We extend a double congratulation to the two doctors now made one, only in one sense, as we learn that each will continue to practice,—and wish them both, singly and collectively, success. The JOURNAL does not exactly catch on to the new racket instituted in this case, of sending out "at home" cards in the name of *Mr.* and *Mrs.* Mathews. When Dr. Byron Robinson married Dr. Lucy Wait they both put M. D., to their name, both "before taking" and "after taking."

---

**The Southern Surgical and Gynecological Association** will hold its seventh annual meeting at Charleston, S. C., November 13, 14 and 15, inst. A general invitation to attend is extended to the medical profession. [The President, Dr. Cornelius Kollock, of Cheraw, S. C., was a brother-in-law of our late lamented Bishop Gregg, and the Bishop's talented son, Dr. C. K. Gregg, whose untimely death, some four years ago, is still lamented by a large circle of friends, was named for him.]

The programme arranged for this meeting is an unusually attractive one, and the cream of the medical talent will participate. We observe in the list of papers one by Joseph Price, one by

Jno. A. Wyeth, Engelman, J. B. Murfree, McMurtry, Lydston, Matas, Wathen, Holmes, J. S. B., and a host of other big guns. Cheap rates have been secured, and there will be a large attendance. For further information address Dr. W. E. B. Davis, Secretary, Birmingham, Ala.

---

**Double Femoral Herniotomy.**—Dr. S. E. Milliken, N. Y. (*La Revista Med. Quirurg.*,) reports a case of double femoral herniotomy at the advanced age of 64 years. Deep sutures of kangaroo tendon were used to close the crural canal, while catgut was employed for bringing together the skin wounds. The dressings were changed the first time on the tenth day, when union was found complete, and the skin sutures absorbed. The highest elevation of temperature was 101° F., which occurred within forty-eight (48) hours and was attributed to the shock of the operation.

CONCLUSIONS:—1. Age is no contra-indication to the employment of the radical cure of hernia.

2. Asepsis and antisepsis should be carefully observed.

3. Even in cases of strangulation the radical cure should be attempted, if the condition of the patient warrants the delay.

4. When the truss becomes a source of annoyance, or if the hernia is difficult to retain, the operation should be performed without delay and before strangulation occurs.

---

**Mississippi Valley Medical Association.**—The twentieth annual meeting of this well-known organization will be held, as previously announced, at Hot Springs, Ark., November 20, 21, 22 and 23, 1894.

The interest and enthusiasm manifested in all parts of the country concerning the meeting in November is certainly remarkable. The fact that Hot Springs is to be the place of meeting will probably be an inducement for many to attend. From the large number of favorable responses to his preliminary announcement, the Secretary feels justified, even thus early, in predicting an attendance double that of any previous meeting of the Association.

Let every doctor who can possibly leave home for a few days go to Hot Springs in November. Let him take his family and his friends, and not only a profitable meeting, but a royal good time, will reward him for the exertion.

FREDERICK C. WOODBURN, Secretary.

399 College Avenue, Indianapolis.

**The College of Physicians of Philadelphia** announces that the next award of the Alvarenga Prize, being the income for one year of the bequest of the late Señor Alvarenga, and amounting to about one hundred and eighty dollars, will be made on July 14, 1895, provided that an essay deemed by the Committee of Award to be worthy of the prize shall have been offered.

Essays intended for competition may be upon any subject in Medicine, but cannot have been published, and must be received by the Secretary of the College on or before May 1, 1895.

Each essay must be sent without signature, but must be plainly marked with a motto and be accompanied by a sealed envelope having on its outside the motto of the paper and within it the name and address of the author.

It is a condition of competition that the successful essay or a copy of it shall remain in the possession of the College; other essays will be returned upon application within three months after the award.

The Alvarenga Prize for 1894 has been awarded to Dr. G. E. de Schweinitz, of Philadelphia, for his essay entitled: "Toxic Amblyopias."

CHARLES W. DULLES, Secretary.

---

**Questions.**—Questions, idle questions, I know not what they mean; questions from the depths of some infernal ignorance or stupidity—to paraphrase Tennyson—are often submitted to this office, to answer some of which would be difficult, some easy. As a sample of the very numerous interrogatories submitted to ye editor, we will mention that not long ago a superintendent of schools in a certain county in Texas asked (without stamp for reply) if we thought a half hour recess at noon for lunch would be prejudicial to the children's health? We replied: Not, if the children were kept in, and not allowed to eat the lunch; that under *no* circumstances should they be allowed to run about out doors and play, or otherwise get any fresh air in their lungs, or rest their limbs and back. In some insurance blanks there are the silliest questions asked: for instance, if the examiner is of the opinion that applicant has a predisposition to cough,—or—sneeze.

In a certain company's blank there is the question, whether the applicant had any predisposition to accidents; an examiner, a friend of ours, showed us an application which he had just filled, and the following are the questions and answers; applicant had been bitten in the foot by a spider:

10 (*a*). Was this accident * * * caused directly or indirectly in consequence of disease or bodily infirmity, or was the disability lengthened thereby? (*b*) or has the claimant any bodily infirmity which renders him liable to accidental injuries, or that would retard recovery therefrom?

Ans. (*Give full particulars.*) The accident was unavoidable. He has no infirmity which predisposes him to spider spite, and has no infirmity which prolonged a recovery.

11. How do you understand he was injured? Ans. By the spider, in his own artful way, getting down into B.'s sock and biting his leg.

---

**Just as We Expected.**—Secretary West has taken exceptions to our criticism of his work, and imputes it to improper motives. He has written a letter to the *Texas Sanitarian*, which will appear in that publication for November. In this letter, while ostensibly correcting certain alleged mistakes made in the *Texas Sanitarian's* review of the Transactions, he takes occasion to rap us pretty severely. He says, "there is one member whose approbation I can never hope to gain; I refer to Dr. Daniel."

Now, this is very unfair and unjust. We can assure him that he can never gain our approbation so long as he is so extravagant in the issue of the yearly volume; but we have always commended his work when we thought it commendable. The doctor seems to think that the JOURNAL holds him responsible for the falling off in membership; whereas it is distinctly stated that "the State is too large, and doctors live too far apart ever to hope to enroll and hold more than a certain per cent. as members." We know that for some reason members have dropped out by the score. During 1889 and 1890 there were 506 members carried on the roll; there are now 354.

We do not undertake to reply to the doctor's letter before it is published, but only write this to show the *raison d'étre* of the editorial in this issue headed "Transactions and the Association."

---

**Holiday Excursions to the South-East.**—On December 20th, 21st and 22nd, 1894, the International Route will as usual, have on sale Holiday Excursion tickets to South-Eastern States, including St. Louis, Louisville, Cincinnati, Memphis and New Orleans, at rate of one fare for round trip, tickets limited to 30 days for return. Call on nearest ticket agent for full information.

D. J. PRICE, A. G. P. A.

### Hygiene of the Young in Schools.

Dr. J. Chalmers Cameron, Professor of Obstetrics and Diseases of Children, McGill University, Montreal, read before the American Public Health Association a paper on the above subject. The paper was a distinct arraignment of school boards for neglecting to provide hygienic appliances. The lecturer pointed out that some thirty years ago it was remarked by Mr. Herbert Spencer that the first requisite of success in life is to be a good animal, and that to be a nation of good animals, is the first condition to national prosperity. As the nation is an aggregation of individuals, the history of the nation is in a certain sense the history of its individual merits. Its strength, progress and development depended upon the strength, progress and development of its members; therefore, other things being equal, that nation will be most prosperous which secures the highest development for its members. From the age of four or five up to fifteen or sixteen, the period of active growth and development, most children are at school, being educated and trained for their life work. If the schools fulfil their important functions well, and turn out their scholars good animals, well equipped for the battle of life, the first condition of national prosperity will have been attained; but in whatever degree they fail to secure the best results, in the same degree will they hinder national progress.

It seems, therefore, peculiarly fitting that the American Public Health Association should examine carefully the methods of the public schools, and inquire whether the best possible is being done, and whether sufficient attention is being paid to the all-important matter of hygiene. However we may theorize as to the nature of man, we can at least distinguish two essential parts, mind and body; and however we may speculate as to their essence and mode of union, we know at least that all life long they are linked together for weal or for woe—they develop together, mature together, decay together, ever dependent upon each other, reacting upon each other, sympathizing with each other, suffering with each other. When we strengthen the body, we invigorate the mind; when we starve and neglect the body, we starve and enfeeble the mind. It follows, therefore, that for the proper development of the individual, the body must be considered and cared for, as well as the mind. According to nature's plan, body and mind develop simultaneously, not alternately. While bone and muscle, nerve and gland are growing and specializing, the child is busy observing, testing, comparing, gaining a knowledge of his environment, and learning to reason and think. So the process goes on; but by and by the child is sent to school. Is the same plan of development continued? Do our school boards realize that education should look to the physical as well as the mental needs of their scholars, and that strong bodies are as essential to success in life as well-stored minds?

When we look at the curriculum of our schools, we find no lack of studies; perhaps the courses are too extensive, and too much is being attempted. We find that the scholars are carefully graded and arranged in various forms and classes; that their work is thoroughly systematized and that they are taught and examined *secundum artem*. All are cared for, none are overlooked. But in how many schools is adequate attention paid to the physique of the scholars? In how many is their physical condition examined and studied? Before they are promoted to a higher form they must obtain a certain percentage of marks in their examination, and demonstrate their ability to undertake more advanced work. But is there ever a question as to their physical ability for the new work? At the end of the year they are examined to determine their scholastic proficiency. Is there ever a question as to how the body has fared meanwhile? Some schools have playgrounds and give a recess presumably for play, but play is optional—the children may play or not as they please—there is no grading, no direction or supervision. Some schools have a gymnasium, but in how many is there a competent instructor to examine the scholars and grade or supervise their work? What sort of progress would there be in a school if the scholars were allowed to choose whatever studies they pleased, go into whatever classes they pleased, and study or not as they pleased? And in like manner what sort of bodily development can be expected when the arrangements for physical training are so crude and unsatisfactory? In respect to physical culture our educational system is sadly deficient. Childhood is the time to detect and prevent such deformities as spinal curvature and pelvic deformity.

Not only should children be taught in school how to stand, sit and walk, but more important still, they should be taught how to breathe. In the larger cities a valuable addition to the educational staff would be an inspector of physical culture. School desks are responsible for a good deal of deformity. The desks and seats are of uniform height, while the pupils are of various sizes. If too tall the children must stoop; if too short they must reach up somehow. No matter whether the spine is curved or the shoulder raised, the chest is compressed. Could not there be, asked the doctor, some simple arrangement of raising and lowering the desks and seats like a piano stool? The doctor considered the bar-bell exercise as most generally applicable to school work. Physical exercise in schools should aim to cultivate the habit of sitting, walking and breathing properly. A child that sits improperly is apt to stand and walk improperly. If the bad habit is not corrected, more or less permanent spinal curvature is apt to result.

Much has been done in investigating the causes of disease and preventing the spread of infection. Can we not go a step further and develop in children strong bodies which will resist the inroads of disease? Bacteriology has taught us that many diseases are directly traceable to the action of microbes introduced into the body from without. The seed, the infective microbe, is the one factor; the suitable soil, the debilitated body is the other factor. Dazzled by the brilliant discoveries of bacteriology, have we not overlooked somewhat the necessity of rendering the soil unsuitable for the growth of the seed by developing the existing powers of the human body? If we attack the problem from both sides, its solution will be easier and more satisfactory. We live in an age of restless activity; now, more than ever, is there need for strong physical frames to bear up in the ever increasing struggle of life. Now, more than ever, are men and women breaking down in middle life, their usefulness cut short when they have become most valuable to society and the State. Now, more than ever is the battle to the strong and staying power is essential.—*Journal Amer. Med. Assn.*

